# Aromatherapy Massage vs. Foot Reflexology on the Severity of Restless Legs Syndrome in Female Patients Undergoing Hemodialysis

**DOI:** 10.3390/geriatrics6040099

**Published:** 2021-10-11

**Authors:** Mahbobeh Ghasemi, Nahid Rejeh, Tahereh Bahrami, Majideh Heravi-Karimooi, Seyed Davood Tadrisi, Mojtaba Vaismoradi

**Affiliations:** 1 Department of Nursing, Faculty of Nursing and Midwifery, Shahed University, Tehran 3319118651, Iran; mahbubeghasemi110@gmail.com (M.G.); reje@shahed.ac.ir (N.R.); btahereh@rocketmail.com (T.B.); heravi@shahed.ac.ir (M.H.-K.); 2 Trauma Research Center, Faculty of Nursing, Baqiyatallah University of Medical Sciences, Tehran 1435916471, Iran; sdt1343@gmail.com; 3 Faculty of Nursing and Health Sciences, Nord University, 8049 Bodø, Norway

**Keywords:** aromatherapy, hemodialysis, reflexology, restless legs syndrome

## Abstract

This study aimed to compare the effects of reflexology and aromatherapy massage on the severity of restless legs syndrome (RLS) in 105 female patients undergoing hemodialysis. A randomized placebo-controlled clinical trial was conducted in a hemodialysis center with 48 beds in a high turnover hospital in an urban area of Iran. Intervention groups received reflexology (*n* = 35) and aromatherapy massage using lavender essential oil (*n* = 35) for 24 sessions, and the placebo group (*n* = 35) received simple foot massage. The restless legs syndrome rating scale was used to assess RLS severity in the groups before the intervention and after 4 and 8 weeks of the interventions. Results obtained by the mixed model analysis 3 * 3 (3 groups * 3 times) revealed the significant effect of time, group, and the time–group interrelationship (*p* = 0.001). Aromatherapy massage reduced the RLS severity, but reflexology did not appear to cause any significant reduction in it. Therefore, we suggest that aromatherapy massage be incorporated into routine care for relieving the ailment and suffering of patients undergoing hemodialysis.

## 1. Introduction

Patients with end-stage renal disease (ESRD) undergo hemodialysis until kidney transplantation becomes an option. However, hemodialysis is accompanied by complications in the nervous system such as sensorimotor neuropathy [1]. Restless legs syndrome (RLS), as a common sensorimotor disorder, is characterized by the patient’s complaint of a strong irresistible urge to move their limbs [2,3]. It has been reported that 30–50% of patients with ESRD and especially patients undergoing hemodialysis suffer from RLS [4,5]. This sensorimotor disturbance can considerably influence patients’ quality of life through impairing their psychological and physiological health conditions and daily life activities [6,7,8].

Different treatment options such as exercise [9], pneumatic compression [10], and near-infrared light [11] can be used to relieve the suffering and pain of patients with RLS. Massage therapy also is considered a suitable option to modulate RLS symptoms. Several speculations about the mechanism of massage therapy in the treatment of RLS have been suggested. For instance, massage can stimulate the cerebral cortex and increase dopamine generation [12]. Additionally, massage therapy can improve tendinous and muscular elasticity and consequently relieve RLS symptoms [13]. Reflexology is one type of massage that applies pressure to specific reflex points on the feet. The stimulation of these reflex points provokes the nervous and endocrine systems [4,14]. Reflexology can also boost the secretion of serotonin, endorphin, and cortisol, which are crucial for the nervous system’s function [15]. The effect of reflexology on RLS is not well documented. However, it has been suggested that the stimulation of reflex zones during reflexology massage increases the brain’s blood oxygen and also leads to immediate hemodynamic responses [16]. Moreover, the muscular relaxant effect of reflexology can support the effectiveness of reflexology on the reduction in RLS symptoms [17].

An alternative strategy is the combination of massage and aromatherapy [18]. Aromatherapy massage initiates amygdala and hippocampus in the limbic system of the brain and leads to physical, emotional, and mental improvements [19]. Essential oils such as lavender (*Lavendula stoechas*) is administrated to skin through massage to provide sedative and muscular relaxant, anxiolytic, antidepressant, neuroprotective, and anti-inflammatory effects [20,21,22]. Different studies have considered lavender as the facilitator of hemodialysis complications such as pain, fatigue, anxiety, and sleep quality [23,24,25,26,27]. Nevertheless, a few studies have compared the effects of reflexology and aromatherapy massage on RLS symptoms [22]. In addition, to our knowledge, the effects of reflexology and aromatherapy massage on RLS among patients undergoing hemodialysis have not been compared. Therefore, this study aimed to compare the effects of reflexology and aromatherapy massage on the severity of RLS in female patients undergoing hemodialysis. Since these two types of massage have been effective in the alleviation of hemodialysis complications, they may also alleviate the severity of RLS. Comparison of aromatherapy massage and reflexology can help healthcare providers with the selection of the best approach in dealing with RLS. This study hypothesis was as follows: aromatherapy massage and foot reflexology massage have equal effects on the improvement of the severity of RLS symptoms among patients undergoing hemodialysis.

## 2. Materials and Methods

### 2.1. Design

**Figure 1:**
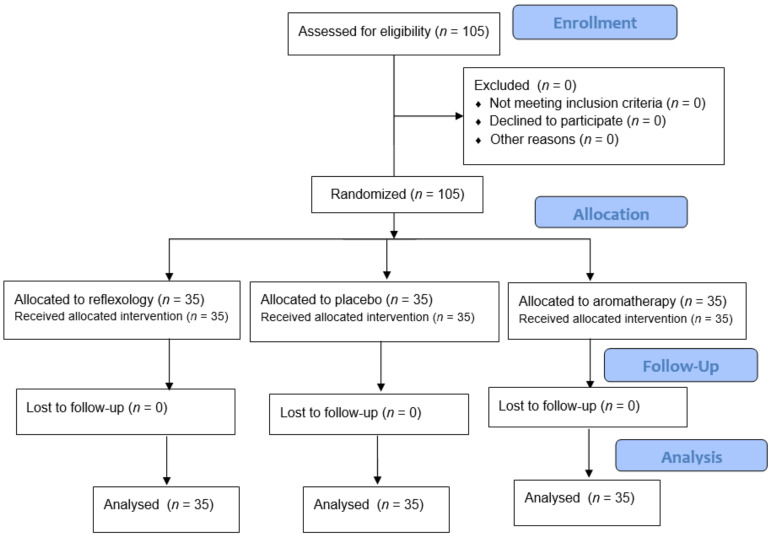
The research process according to the Consolidated Standards of Reporting Trials (CONSORT) flow diagram.

This randomized placebo-controlled clinical trial with a before-and-after design involved 105 female patients undergoing hemodialysis. They were assigned to reflexology massage (*n* = 35), aromatherapy massage (*n* = 35) and placebo (*n* = 35) groups. The outcome measure was the comparison of the effects of reflexology and aromatherapy massage on RLS symptoms before and after the interventions (Figure 1). The research protocol was registered on the website of Registry of Clinical Trials (decree code: IRCT201612067529N11).

### 2.2. Setting and Sampling

This research was carried out in a hemodialysis center with 48 beds in a hospital in an urban area of Iran from October 2018 to April 2019. Female patients with ESRD and undergoing hemodialysis were chosen to participate. To prevent the effect of gender bias, only female patients were recruited.

Using a sampling formula, given the results of a previous study on patients with RLS hospitalized in critical care units [28], 95% confidence interval and 80% power, the necessary sample size in each group was estimated to be 33 patients. However, given the possibility of sample dropout [29], 35 patients were recruited into each group.

After obtaining required permissions, a convenience sample of female patients undergoing hemodialysis was chosen, with no patient declining to participate. A system of sealed envelopes was used for the random assignment of eligible participants into the groups with each envelope assigned to a specific group. The sampling process continued until a sufficient number of participants were recruited into each group. The second author (N.R.) generated the random allocation sequence, and the first author (M.G.) enrolled the participants in the groups.

### 2.3. Blinding

The smell of lavender oil made it impossible to blind the participants. However, the data analyst (S.D.T.) was unaware of the groups’ assignment to avoid bias.

### 2.4. Eligibility Criteria

Inclusion criteria were female gender, diagnosis of RLS by a physician, and the presence of four criteria of the International RLS study group (IRLSSG) [30]: age above 18 years; not taking anxiolytics and sedative medications at least last 4 h before the interventions; having no history of alternative and complementary care in the last 48 h; absence of foot ulcers; having no history of drug addiction, asthma, eczema, and allergy; undergoing hemodialysis three times a week for 6 months and each session lasting for 3–4 h; having no history of mental or physical disability including peripheral neuropathy or vascular problems in legs; being able to walk on the feet.

Refusal to complete the intervention sessions, hemodynamic instabilities during the interventions, and any need for emergency treatments were considered exclusion criteria.

### 2.5. Measures

The demographic characteristics form consisted of questions about the participants’ age, education level, marital status, employment status, family history of RLS, duration of hemodialysis, and smoking habits.

The severity of RLS was assessed using the RLS rating scale. It consists of 10 items with five options from 0 to 4 and a total score ranging from 0 to 40, with a higher score indicating the greater severity of RLS. A score less than 10 was considered mild RLS, 11–20 moderate, 21–30 severe, and above 31 very severe. The reliability of the RLS rating scale was assessed through the calculation of Cronbach’s alpha coefficient and was 0.97. The content validity index of the RLS rating scale was 0.87 [31].

The RLS rating scale was completed for all groups by a staff nurse who was unaware of the group assignments before the first intervention session and after 4 and 8 weeks of the interventions.

### 2.6. Interventions

#### 2.6.1. Reflexology

The female researcher (M.G.) who had received education about foot reflexology performed the interventions. The participants received foot reflexology three times a week for 8 consecutive weeks. The participants were placed in the semi-fowler position with their feet positioned at the therapist’s chest level. Relaxing techniques consisting of a general foot massage with six drops of almond oil on each foot was applied for five minutes. The massage consisted of a mild pressure of the therapist’s hands on the patient’s foot and pressing of the foot with the fist. The pressure force was adjusted according to the patient’s tolerance and feeling of comfort. When the participants felt warm on the foot or the therapist’s fingers turned white, the pressure was stopped to prevent the feeling of pain and discomfort. Next, reflexology was applied 15 min for each foot (30 min on both feet) on reflex points including hypothesis, thyroid, parathyroid, pancreas, adrenal glands, and solar plexus [32], starting with the right foot. The intervention was performed three times a week for 8 consecutive weeks. On average, 2–4 patients received massages each day.

#### 2.6.2. Aromatherapy Massage

Aromatherapy massage consisting of reflexology using lavender essential oil was applied by the same researcher (M.G.). The formula of this essential oil was linalool (27.11%) and linalyl acetate (23.33%) in the ratio of 3:3:2:2 mL in 100 mL of coconut carrier oil. The lavender essential oil was chosen after consultation with experts at the department of pharmacology of the university where the corresponding author works. Relaxing techniques consisted of a mild pressure of the therapist’s hands on the patient’s foot and also pressing the foot with the fist. The pressure was adjusted according to the patient’s tolerance and feeling of comfort. The same reflex points on the foot were massaged using 10 drops of lavender essential oil. The duration of the aromatherapy massage was 15 min on each foot, performed three times a week for 8 consecutive weeks.

No negative consequences or side effects relating to the aromatherapy massage and reflexology interventions were reported.

#### 2.6.3. Placebo

Relaxing techniques similar to the intervention groups were applied on each foot using almond oil. The same therapist performed the massage without the stimulation of the reflex points on the foot and with a similar duration as the intervention groups. The RLS rating scale was also filled out by the participants in this group.

### 2.7. Ethical Considerations

This research was conducted with the consideration of ethical principles in accordance with the Declaration of Helsinki, 1995, revised in 2001. The study’s aim and method were explained to each participant. Those willing to take part in our research signed an informed consent form. They were informed of the possibility of withdrawal from the study at any time without any effect on their care. Codes rather than names were used to identify the patients and preserve their anonymity as well as the confidentiality of the data. A nephrologist was available to intervene if any negative consequence of the interventions was observed.

### 2.8. Data Analysis

Data were analyzed using descriptive (frequency, percentage, mean, and standard deviation) and inferential statistics (ANOVA, Chi-squared test, Fisher’s exact test) via SPSS (SPSS Inc., Chicago, IL, USA). The Kolmogorov–Smirnov test examined the normal distribution of data. The mixed model analysis 3 * 3 (3 groups * 3 times) was performed to analyze the effect of time, group, and the time–group interrelationship.

## 3. Results

### 3.1. Demographic Characteristics

The participants had a mean age of 50.46 ± 0.86 years. According to the one-way ANOVA test, the groups were homogenous in terms of age (*p* = 0.09) but not educational level (*p* = 0.001). The Chi-square test showed no statistically significant differences between the groups in terms of the marital status, employment status, family history of RLS, duration of hemodialysis, and smoking habits (*p* > 0.05). (Table 1).

### 3.2. Effects of the Interventions on RLS Severity

The mixed model analysis 3 * 3 (3 groups * 3 times) helped assess the effect of time, group, and the time–group interrelationship on RLS severity. The statistical significance of time indicated that RLS severity was significantly different during the study time intervals. Moreover, the interventions made significant changes in RLS severity (*p* < 0.05). The interrelationship between time and groups also revealed significant differences between the research groups during the time intervals (*p* = 0.001) (Table 2).

The comparison between different time intervals showed that RLS severity was not significantly different before and after 8 weeks of the interventions (*p* > 0.05). Comparison between the research groups indicated that RLS severity was significantly different in the reflexology and aromatherapy massage groups (*p* < 0.05). However, the interrelationship between time and intervention showed that only in the aromatherapy massage group, RLS severity was significantly different after 8 weeks of the intervention (*p* = 0.001). (Table 2)

## 4. Discussion

According to our research results, RLS severity was only significantly different in the aromatherapy massage group during the time intervals. Therefore, our research hypothesis that reflexology and aromatherapy massage had similar effects on RLS severity was not supported. This can be attributed to some extent to the addition of lavender essential oil to reflexology massage in the aromatherapy massage group. It is believed that essential oils include analgesic components that can influence neurotransmitters such as dopamine and serotonin, and noradrenaline receptor sites in the brain. The interaction of touch with sensory fibers in the skin and the rate of absorption of essential oils are increased during aromatherapy massage [33].

No study with the same research process and intervention durations was found to be used for the comparison of our findings. However, a randomized controlled trial by Hashemi et al. on the effect of effleurage massage using lavender oil in patients with RLS and undergoing hemodialysis for three weeks showed a reduction in the mean score of RLS [22]. Another study by Nasiri et al. supported the effect of massage using olive oil on the severity of uremic RLS on the first day and one week after finishing massage therapy [34].

Aromatherapy massage is an inexpensive, rapid-acting, and effective intervention for reducing the complications of hemodialysis [35]. Studies by Hassanzadeh et al. and Sentürk and Tekinsoy reported that aromatherapy massage reduced fatigue and anxiety, and improved sleep quality in patients undergoing hemodialysis [36,37].

Data analysis revealed that RLS severity was not different in the reflexology group during the time intervals. Shahgholian et al. compared reflexology and stretching exercises in patients suffering from RLS and undergoing hemodialysis and showed a significant difference in the mean scores of RLS severity between reflexology and control groups. However, a lack of statistically significant differences between these techniques indicated their equal effectiveness [38]. Reflexology can reduce fatigue, pain and cramps, and improve quality of sleep in patients undergoing hemodialysis [39]. Ahmadidarrehsima et al. (2018) reported that reflexology was a quite safe intervention to reduce fatigue in patients undergoing hemodialysis [40].

The main consequence of ESR is chronic and long-term illness, which can reduce the effectiveness of reflexology massage [41]. However, performing reflexology for a long duration or motivating patients to perform self-foot reflexology can help hemodialysis patients to overcome the negative consequences of RLS on their quality of life.

This research is a pioneer of the comparison of the effects of reflexology and aromatherapy massage on the severity of RLS in patients undergoing hemodialysis. The effect of gender bias on the study outcomes was avoided through the recruitment of female patients. Moreover, the duration of follow-up was extended to eight weeks after the interventions. The patients’ awareness of the interventions possibly increased the effects of aromatherapy massage and reflexology, and the nature of the interventions as the smell of lavender oil hindered the blinding of the participants. Further studies on male patients and with a longer follow-up period are suggested for investigating gender differences and the long-term effects of these interventions.

## 5. Conclusions

Aromatherapy massage was shown to be more effective than reflexology on the alleviation of RLS severity in female patients undergoing hemodialysis. Adding an essential oil such as lavender to massage therapy is a safe and inexpensive method that may help relieve RLS symptoms. In the reflexology group, no statistically significant difference was reported during the time intervals. However, further research is required to investigate the effect of reflexology on RLS severity.

## Figures and Tables

**Table 1:** Demographic characteristics of the patients in the groups.

Characteristic	Aromatherapy Massage	Reflexology	Placebo	*p*-Value
Age (year)	48.17 ± 1.65	52.77 ± 1.58	50.45 ± 1.13	0.09
Education level				0.001
Illiterate	3 (2.9%)	9 (8.6%)	8 (7.6%)	
Elementary	24 (22.9%)	29 (27.6%)	13 (12.4%)	
Diploma	3 (2.9%)	2 (1.9%)	14 (33.3%)	
Marital status				1
Married	30 (28.6%)	30 (28.6%)	30 (28.6%)	
Single and widow	5 (4.8%)	5 (4.8%)	5 (4.8%)	
Occupation				0.6
Housewife	34 (32.5%)	35 (33.3%)	34 (32.5%)	
Employed	1 (0.9%)	0 (0%)	1 (0.9%)	
Family history of restless legs syndrome				0.12
Yes	8 (7.6%)	3 (2.9%)	3 (2.9%)	
No	27 (25.7%)	32 (30.5%)	32 (30.5%)	
Duration of hemodialysis	5.07 ± 0.53	5.41 ± 0.48	5.31 ± 0.43	0.49
Smoking				0.81
Yes	2 (1.9%)	2 (1.9%)	1 (0.9%)	
No	33 (31.4%)	33 (31.4%)	34 (32.4%)	

**Table 2:** Comparison of the mean scores of the severity of RLS at different time intervals between the group.

Time	Group	Mean ± SD		
Before the intervention	Reflexology	19.77 ± 3.049		
	Aromatherapy massage	21.71 ± 4.515		
	Placebo	20.43 ± 4.565		
After 4 weeks of the intervention	Foot Reflexology	18.60 ± 5.192		
	Aromatherapy	17.06 ± 5.179		
	Placebo	20.54 ± 3.071		
After 8 weeks of the intervention	Foot Reflexology	16.80 ± 5.357		
	Aromatherapy	13.20 ± 4.880		
	Placebo	19.51 ± 2.904		
Tests of Fixed Effects				Estimates of Fixed Effects
Source	DF	F	Sig	
Time	2102	36.89	0.001	a vs. c SE = 0.87, t = 1.04, *p* = 0.30 b vs. c SE = 0.60, t = 1.69, *p* = 0.09
Group	2102	5.70	0.004	Reflexology vs. Placebo SE = 1.07, t = −2.51, *p* = 0.01 Aromatherapy vs. Placebo SE = 1.07, t = −5.86, *p* = 0.001
Time*Group	4102	10.06	0.001	Reflexology *^,a^ vs. Placebo *^,a^ SE = 1.24, t = 1.65, *p* = 0.10 Aromatherapy *^,a^ vs. Placebo *^,a^ SE = 1.24, t = 6.12, *p* = 0.001 Foot Reflexology *^,b^ vs. Placebo *^,a^ SE = 0.85, t = 0.90, *p* = 0.37 Aromatherapy *^,b^ vs. Placebo *^,a^ SE = 0.85, t = 3.30, *p* = 0.001

^a^: Before the intervention. ^b^: After 4 weeks of the intervention. ^c^: After 8 weeks of the intervention. *: Interaction.

## Data Availability

All data generated or analyzed during this study are included in this published article.

## References

[1] Lin X.-W., Zhang J.-F., Qiu M.-Y., Ni L.-Y., Yu H.-L., Kuo S.-H., Ondo W.G., Yu Q., Wu Y.-C. (2019). Restless legs syndrome in end stage renal disease patients undergoing hemodialysis. BMC Neurol..

[2] Sateia M.J. (2014). International Classification of Sleep Disorders-Third Edition: Highlights and modifications. Chest.

[3] Guo S., Huang J., Jiang H., Han C., Li J., Xu X., Zhang G., Lin Z., Xiong N., Wang T. (2017). Restless Legs Syndrome: From Pathophysiology to Clinical Diagnosis and Management. Front. Aging Neurosci..

[4] Kim J.-M., Kwon H.-M., Lim C.S., Kim Y.S., Lee S.-J., Nam H. (2008). Restless Legs Syndrome in Patients on Hemodialysis: Symptom Severity and Risk Factors. J. Clin. Neurol..

[5] Jafari M., Rafie S., Azizi M., Bahadoram M., Jafari S. (2016). Restless legs syndrome in hemodialysis patients. Saudi J. Kidney Dis. Transplant..

[6] Lee J. (2008). A Review of Restless Legs Syndrome in Patients on Hemodialysis. Kidney.

[7] Selcuk N., Kutlu R., Sayin S., Kal O. (2018). Restless legs syndrome and quality of life in chronic hemodialysis patients. Niger. J. Clin. Pract..

[8] Yıldız A. (2018). Assessment of cardiac autonomic functions by heart rate variability in patients with restless legs syndrome. Turk Kardiyol. Dern. Ars..

[9] Lakasing E. (2008). Exercise beneficial for restless legs syndrome. Practitioner.

[10] Lettieri C.J., Eliasson A.H. (2009). Pneumatic Compression Devices Are an Effective Therapy for Restless Legs Syndrome: A prospective, randomized, double-blinded, sham-controlled trial. Chest.

[11] Mitchell U.H., Myrer J.W., Johnson A.W., Hilton S.C. (2010). Restless legs syndrome and near-infrared light: An alternative treatment option. Physiother. Theory Pract..

[12] Mitchell U.H. (2011). Nondrug-related aspect of treating Ekbom disease, formerly known as restless legs syndrome. Neuropsychiatr. Dis. Treat..

[13] Draper D.O., Tessier D.G. (2005). Sports Massage: An Overview. Athl. Ther. Today.

[14] Bahrami T., Rejeh N., Heravi-Karimooi M., Vaismoradi M., Tadrisi S., Sieloff C.L. (2017). Aromatherapy massage versus reflexology on female elderly with acute coronary syndrome. Nurs. Crit. Care.

[15] Embong N.H., Soh Y.C., Ming L.C., Wong T.W. (2015). Revisiting reflexology: Concept, evidence, current practice, and practitioner training. J. Tradit. Complement. Med..

[16] Huang H., Chen K., Kuo S., Chen I. (2021). Can foot reflexology be a complementary therapy for sleep disturbances? Evidence appraisal through a meta-analysis of randomized controlled trials. J. Adv. Nurs..

[17] Özdemir G., Ovayolu N., Ovayolu Ö. (2013). The effect of reflexology applied on hemodialysis patients with fatigue, pain and cramps. Int. J. Nurs. Pract..

[18] Ali B., Al-Wabel N.A., Shams S., Ahamad A., Khan S.A., Anwar F. (2015). Essential oils used in aromatherapy: A systemic review. Asian Pac. J. Trop. Biomed..

[19] Metin Z.G., Donmez A.A., Izgu N., Ozdemir L., Arslan I.E. (2017). Aromatherapy Massage for Neuropathic Pain and Quality of Life in Diabetic Patients. J. Nurs. Sch..

[20] Gedney J.J., Glover T.L., Fillingim R. (2004). Sensory and Affective Pain Discrimination after Inhalation of Essential Oils. Psychosom. Med..

[21] Firoozeei T.S., Feizi A., Rezaeizadeh H., Zargaran A., Roohafza H.R., Karimi M. (2021). The antidepressant effects of lavender (Lavandula angustifolia Mill.): A systematic review and meta-analysis of randomized controlled clinical trials. Complement. Ther. Med..

[22] Hashemi S.H., Hajbagheri A., Aghajani M. (2015). The Effect of Massage with Lavender Oil on Restless Leg Syndrome in Hemodialysis Patients: A Randomized Controlled Trial. Nurs. Midwifery Stud..

[23] Özdemir S.T., Akyol A. (2021). Effect of inhaler and topical lavender oil on pain management of arteriovenous fistula cannulation. J. Vasc. Access.

[24] Kang H.-J., Nam E.S., Lee Y., Kim M. (2019). How Strong is the Evidence for the Anxiolytic Efficacy of Lavender? Systematic Review and Meta-analysis of Randomized Controlled Trials. Asian Nurs. Res..

[25] Karadag E., Bağlama S.S. (2019). The Effect of Aromatherapy on Fatigue and Anxiety in Patients Undergoing Hemodialysis Treatment: A randomized controlled study. Holist. Nurs. Pract..

[26] Muz G., Taşcı S. (2017). Effect of aromatherapy via inhalation on the sleep quality and fatigue level in people undergoing hemodialysis. Appl. Nurs. Res..

[27] Ajorpaz N.M., Rahemi Z., Aghajani M., Hashemi S.H. (2020). Effects of glycerin oil and lavender oil massages on hemodialysis patients’ restless legs syndrome. J. Bodyw. Mov. Ther..

[28] Habibzade H., Khalkhali H., Ghaneii R. (2011). Study of the relationship between restless legs syndrome and sleep disturbance among patients in critical care units. Iran J. Crit. Care Nurs..

[29] Babashahi M., Kahangi L., Babashahi F., Fayazi S. (2012). Comparing the Effect Of Massage Aromatherapy and Massage on Anxiety Level Of the Patients in the Preoperative Period: A clinical trial. Evid. Based Care.

[30] International RLS Study Group (IRLSSG) Diagnostic Criteria. http://irlssg.org/diagnostic-criteria.

[31] Atkinson M., Allen R., DuChane J., Murray C., Kushida C., Roth T. (2004). Validation of the Restless Legs Syndrome Quality of Life Instrument (RLS-QLI): Findings of a Consortium of National Experts and the RLS Foundation. Qual. Life Res..

[32] Karatas N., Dalgic A.I. (2020). Effects of reflexology on child health: A systematic review. Complement. Ther. Med..

[33] Buckle J. (2015). Clinical Aromatherapy-E-Book: Essential Oils in Healthcare.

[34] Nasiri M., Abbasi M., Khosroabadi Z.Y., Saghafi H., Hamzeei F., Amiri M.H., Yusefi H. (2019). Short-term effects of massage with olive oil on the severity of uremic restless legs syndrome: A double-blind placebo-controlled trial. Complement. Ther. Med..

[35] Bouya S., Ahmadidarrehsima S., Badakhsh M., Balouchi A., Koochakzai M. (2018). Effect of aromatherapy interventions on hemodialysis complications: A systematic review. Complement. Ther. Clin. Pract..

[36] Hassanzadeh M., Kiani F., Bouya S., Zarei M. (2018). Comparing the effects of relaxation technique and inhalation aromatherapy on fatigue in patients undergoing hemodialysis. Complement. Ther. Clin. Pract..

[37] Şentürk A., Kartın P.T. (2018). The Effect of Lavender Oil Application via Inhalation Pathway on Hemodialysis Patients’ Anxiety Level and Sleep Quality. Holist. Nurs. Pract..

[38] Shahgholian N., Jazi S.K., Karimian J., Valiani M. (2016). The effects of two methods of reflexology and stretching exercises on the severity of restless leg syndrome among hemodialysis patients. Iran. J. Nurs. Midwifery Res..

[39] Unal K.S., Akpinar R.B. (2016). The effect of foot reflexology and back massage on hemodialysis patients’ fatigue and sleep quality. Complement. Ther. Clin. Pract..

[40] Ahmadidarrehsima S., Mohammadpourhodki R., Ebrahimi H., Keramati M., Dianatinasab M. (2018). Effect of foot reflexology and slow stroke back massage on the severity of fatigue in patients undergoing hemodialysis: A semi-experimental study. J. Complement. Integr. Med..

[41] Kliegel E. (2018). Holistic Reflexology: Essential Oils and Crystal Massage in Reflex Zone Therapy.

